# THE ROLE OF SOCIODEMOGRAPHIC FACTORS IN COMMUNITY-BASED REHABILITATION USE AFTER HIP ARTHROPLASTY

**DOI:** 10.1093/geroni/igae098.2852

**Published:** 2024-12-31

**Authors:** Allyn Bove, Christopher Bise, Lauren Terhorst, Kelli Allen, Jared Magnani, Anthony Delitto, Janet Freburger

**Affiliations:** University of Pittsburgh, Pittsburgh, Pennsylvania, United States; University of Pittsburgh, Pittsburgh, Pennsylvania, United States; University of Pittsburgh, Pittsburgh, Pennsylvania, United States; Durham VA Health Care System, Durham, North Carolina, United States; University of Pittsburgh, Pittsburgh, Pennsylvania, United States; University of Pittsburgh, Pittsburgh, Pennsylvania, United States; University of Pittsburgh, Pittsburgh, Pennsylvania, United States

## Abstract

Community-based rehabilitation in the home health and outpatient settings is an important element of recovering function after total hip arthroplasty (THA). While there is ample evidence confirming sociodemographic differences in THA rates and short-term outcomes (e.g., post-operative complications), it is unclear whether there are differences in use of rehabilitation after THA. We analyzed 5 years of data (2017-2021) from a large health system on patients who received rehabilitation in the home health (n=6577) and/or outpatient setting (n=3,094) after THA. We estimated several regression models to examine the separate associations between sex, race, area deprivation, or rural residence with the number of visits and length of care in home health and outpatient settings, adjusting for age, hospital, hospital length of stay, non-home discharge, comorbidities, and marital status. Female sex was associated with greater rehabilitation use for 3 of 4 outcomes: number of home health visits (p<.001), number of outpatient visits (p=.04), and length of outpatient care (p<.001), but not length of home health care. High area deprivation was associated with higher use of home health visits (p<.001), but not length of the episode of home health care or outpatient rehabilitation use. Rural residence was associated with fewer home health visits (p=.04) and shorter outpatient episodes of care (p=.02). Race was not significantly associated with any of the outcomes. Overall, results were mixed, with rural residents generally receiving less post-arthroplasty rehabilitation, and women and those residing in more-deprived neighborhoods receiving more rehabilitation services.

